# A critical role for ATF2 transcription factor in the regulation of E‐selectin expression in response to non‐endotoxin components of *N* *eisseria meningitidis*

**DOI:** 10.1111/cmi.12483

**Published:** 2015-07-31

**Authors:** M. C. Jacobsen, P. J. Dusart, K. Kotowicz, M. Bajaj‐Elliott, S. L. Hart, N. J. Klein, G. L. Dixon

**Affiliations:** ^1^ Infection, Inflammation and Rheumatology SectionInstitute of Child HealthUniversity College LondonLondonUK; ^2^ Department of BiologyFaculty of ScienceUniversity of ReginaReginaSKCanada; ^3^ Science for Life Laboratory, Clinical Applied ProteomicsSchool of BiotechnologyRoyal Institute of Technology (KTH)SolnaSweden; ^4^ Experimental and Personalised Medicine SectionInstitute of Child HealthUniversity College LondonLondonUK; ^5^ Department of MicrobiologyGreat Ormond Street HospitalLondonUK

## Abstract

Vascular injury is a serious complication of sepsis due to the gram‐negative bacterium *Neisseria meningitidis*. One of the critical early steps in initiating this injury is via the interaction of leucocytes, particularly neutrophils, with adhesion molecules expressed on inflamed endothelium. We have previously demonstrated that both lipopolysaccharide (LPS) and non‐LPS components of meningococci can induce very high levels of expression of the vascular endothelial cell adhesion molecule E‐selectin, which is critical for early tethering and capture of neutrophils onto endothelium under flow. Using an LPS‐deficient strain of meningococcus, we showed that very high levels of expression can be induced in primary endothelial cells, even in the context of weak activation of the major host signal transduction factor [nuclear factor‐κB (NF‐κB)]. In this study, we show that the particular propensity for *N* *. meningitidis* to induce high levels of expression is regulated at a transcriptional level, and demonstrate a significant role for phosphorylation of the ATF2 transcription factor, likely via mitogen‐activated protein (MAP) kinases, on the activity of the E‐selectin promoter. Furthermore, inhibition of E‐selectin expression in response to the *lpxA−* strain by a p38 inhibitor indicates a significant role of a p38‐dependent MAPK signalling pathway in ATF2 activation. Collectively, these data highlight the role that LPS and other bacterial components have in modulating endothelial function and their involvement in the pathogenesis of meningococcal sepsis. Better understanding of these multiple mechanisms induced by complex stimuli such as bacteria, and the specific inflammatory pathways they activate, may lead to improved, focused interventions in both meningococcal and potentially bacterial sepsis more generally.

## Introduction

The gram‐negative bacterium *Neisseria meningitidis* is the leading cause of meningitis and septicaemia worldwide. Despite improvements in treatment and intensive care, overall mortality remains at about 5–10% (Milonovich, 2007). Most fatalities are seen in severe meningococcal septicaemia, which is characterized by extensive vascular damage, capillary leakage, and intravascular thrombosis and shock. The mechanism by which this bacterium is able to induce such severe vascular damage remains incompletely understood. It is known that high levels of bacteraemia and lipopolysaccharide (LPS) are associated with increased vascular damage and poor clinical outcome (Brandtzaeg *et al*., 1989; Hackett *et al*., 2002; Ovstebo *et al*., 2004).

Histopathological and clinical *in vivo* studies on both post‐mortem and tissue biopsy material from cases have consistently demonstrated the presence of meningococci adhering to and invading vascular endothelium associated with a dense inflammatory infiltrate with neutrophils predominating (Harrison *et al*., 2002). We have previously shown *in vitro* that meningococci‐induced endothelial damage is largely due to an increase in neutrophil binding (Klein *et al*., 1996). The adhesion of neutrophils to the vascular endothelium is largely mediated by the expression and function of adhesion molecules on both the neutrophil and the endothelial cell surfaces (Luscinskas and Gimbrone, 1996). Infection with live, wild‐type (WT) *N. meningitidis* leads to a marked increase in E‐selectin, Intracellular Adhesion Molecule‐1 (ICAM‐1) and Vascular Adhesion Molecule‐1 (VCAM‐1) endothelial expressions (Dixon *et al*., 2004). The data also suggest that, in contrast to VCAM‐1 and ICAM‐1 expressions, which closely mirror the LPS content of the WT bacteria, E‐selectin expression induced by meningococci appears to be differentially controlled. Hence, levels of E‐selectin induced by WT meningococci always exceed response to equivalent dose of purified LPS based on the LPS content of bacteria (Dixon *et al*., 1999). Furthermore, an LPS‐deficient mutant (*lpxA‐*) retains the ability to induce high E‐selectin expression, exceeding the maximum level induced by high doses of purified LPS. Although WT bacteria and meningococcal LPS are potent inducers of the transcription factor NF‐κB (nuclear factor‐κB), this level of activation is not observed in response to the *lpxA‐* strain (Dixon *et al*., 2004). Taken together, this suggests that non‐LPS components of meningococci primarily affect signalling pathways, without significant NF‐κB involvement, leading to increased E‐selectin transcription and expression.

To improve our understanding of how LPS‐independent mechanisms contribute to the regulation of E‐selectin expression, we undertook detailed analysis of transcriptional responses and promoter activity that govern E‐selectin expression in primary human endothelial cells. The results obtained indicated that both LPS and non‐LPS components of meningococci act in concert to drive high levels of E‐selectin expression. LPS‐independent mechanisms governing E‐selectin expression included mediation via the activation of the transcription factor Activation Transcription Factor 2 (ATF2), principally involving p38 mitogen‐activated protein (MAP) kinase. The pathophysiological consequences of marked induction of E‐selectin expression leading to increased neutrophil binding and activation most likely contribute to enhanced endothelial damage and severe sepsis. Understanding the mechanisms regulating these processes may lead to novel therapies and improved treatment for meningococcal‐induced septic shock.

## Results

### Differential adhesion molecule surface expression induced by *N* *. meningitidis* H44/76 on endothelial cells is not dependent upon bacterial viability

**Figure 1:**
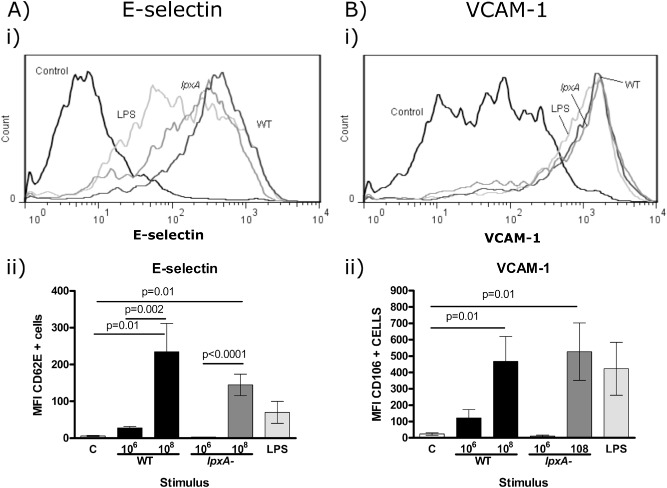
E‐selectin was differentially expressed on HUVEC in response to WT and *lpxA‐* *N* *. meningitidis* *H* *44/76* and meningococcal LPS. HUVEC were stimulated for 5 h with 10^6^ and 10^8^ cfu ml^−1^ fixed WT (dark grey line/black bars) and *lpxA‐* bacteria (dark grey line/dark grey bars) and 10 ng ml^−1^ LPS (light grey line/light grey bars), or media only (Control). Cells were stained for E‐selectin and VCAM‐1 as described in the Experimental procedures section. (A) E‐selectin and (B) VCAM‐1 expression. (i) Representative histograms of E‐selectin and VCAM‐1 expression on HUVEC. (ii) Summary of E‐selectin and VCAM‐1 expression in response to the different stimuli. Results are presented as mean of MFI ± SEM, *n* = 7.

We have previously reported that live *N. meningitidis* can induce vascular adhesion molecule expression and affects E‐selectin expression differentially compared with purified LPS (Dixon *et al*., 2004). In related studies, we observed that live and paraformaldehyde (PFA)‐killed *N. meningitidis* exhibit varying capacities in modulating host immunity (Jones *et al*., 2007), and consequently, we aimed to establish how infection with killed *N. meningitidis* may impact on endothelial E‐selectin expression. Both WT and *lpxA‐* bacteria induced higher levels of E‐selectin compared with the maximum level achievable in response to purified meningococcal LPS (Fig. 1Ai). In contrast, all stimuli were equally potent at inducing VCAM‐1 expression (Fig. 1Bi). We next investigated whether the differential effects on E‐selectin compared to VCAM‐1 induction hold true for different doses of bacteria. As Fig. 1Aii shows, at a lower bacterial dose of 10^6^ cfu ml^−1^, the WT bacteria induced low level expression of both E‐selection and VCAM‐1, whereas infection with the *lpxA‐* bacteria mediated minimal effect on both adhesion molecules expression. At 10^8^ cfu ml^−1^ infectious dose, WT and *lpxA‐* bacteria both induced E‐selectin and VCAM‐1 expression, which was significant when compared to control, uninfected cells (*P* < 0.05) (Fig. 1Aii and Bii). The LPS dose used in these experiments corresponded to the content found in 10^7^ cfu ml^−1^ bacteria (G. Dixon, pers. comm.). Importantly, the LPS dose utilized in the present study led to maximal E‐selectin expression (data not shown). Taken together, the data suggested that multiple, potentially synergistic signals may be operating to differentially affect E‐selection expression in response to LPS and non‐LPS components of the bacteria.

### *N. meningitidis* H44/76 differentially increases E‐selectin but not VCAM‐1 at a transcriptional level

**Figure 2:**
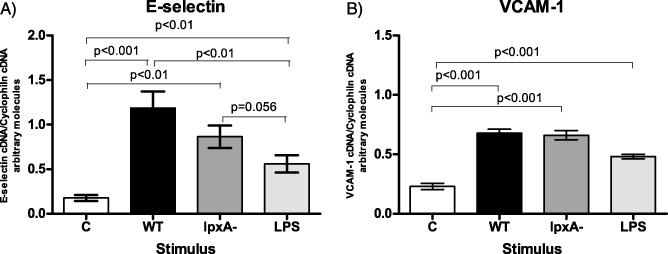
E‐selectin mRNA was differentially expressed in HUVEC in response to fixed WT and *lpxA‐* *N* *. meningitidis* *H* *44/76* and purified LPS, which was not observed in the expression pattern of VCAM‐1 mRNA. HUVEC were stimulated for 4 h with 10^8^ cfu ml^−1^ fixed WT (black bars) and *lpxA‐* (dark grey bars) bacteria or 10 ng ml^−1^ purified LPS (light grey bars), and mRNA levels of (A) E‐selectin and (B) VCAM‐1 were detected using real‐time RT‐PCR. Data were normalized using the *gene induction* method, described in the Experimental procedures section. Results are presented as mean of *gene induction* ± SEM, *n* ≥ 4.

In order to determine if the LPS‐independent mechanisms regulating E‐selectin expression were controlled at the transcriptional level, E‐selectin mRNA expression was determined 4 h post‐stimulation by quantitative real‐time PCR (qPCR). E‐selectin gene expression showed significant increases in response to infection with WT (*P* < 0.001) and *lpxA‐* bacteria (*P* < 0.01) when compared to non‐infected control cells (Fig. 2A). A modest but significant increase of E‐selectin mRNA expression was also observed with purified LPS (*P* < 0.05). WT bacteria induced greater E‐selectin mRNA expression compared with the levels obtained with LPS stimulation (*P* < 0.01); however, this difference was not observed between *lpxA‐* and LPS induced E‐selectin mRNA expression. VCAM‐1 mRNA expression was also detected in response to WT and *lpxA‐* meningococci and purified LPS. Interestingly, VCAM‐1 levels detected were similar in response to all three stimuli (Fig. 2B). Taken together, these data indicated that differential E‐selectin surface expression on endothelium in response to WT bacteria and purified LPS is regulated at the transcriptional level.

### The PDII region of the E‐selectin promoter is responsible for transcriptional activity induced by non‐LPS components of *N* *. meningitidis* H44/76

**Figure 3:**
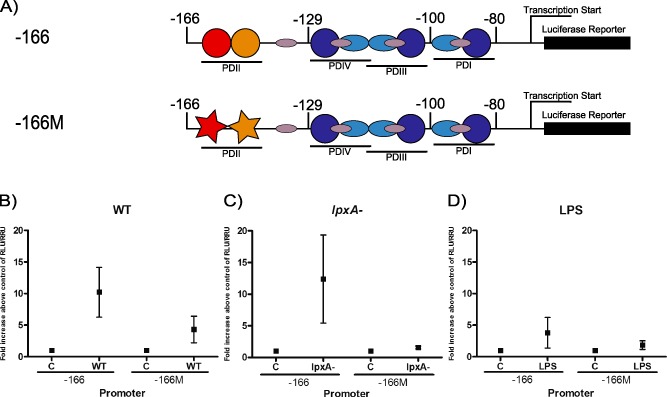
A mutation of an ATF2 site on the E‐selectin promoter reduced promoter activity induced by fixed *N* *. meningitidis* *H* *44/76* and LPS. A. E‐selectin promoter constructs used in this study. (A) The ‐166 promoter construct contains the four PD regions of the E‐selectin promoter. The ‐166M construct contains a mutated PDII region, which is an ATF2 binding site. B–D. HUVEC were transfected with the ‐166 and ‐166M E‐selectin promoter constructs, as described in the Experimental procedures section. Transfected cells were then stimulated with fixed 10^8^ cfu ml^−1^ WT (B) or *lpxA‐* bacteria (C), 10 ng ml^−1^ purified LPS (D), or media only for 5 h. Cells were harvested and luciferase and Renilla activity was detected. Results are presented as mean of fold increase above control of RLU/RRU activity ± SEM, *n* ≥ 3.

The proximal E‐selectin promoter contains four transcription factor binding regulatory regions designated as PD I–IV, all known to contribute to E‐selectin promoter activity (Whitley *et al*., 1994; Collins *et al*., 1995). PDI, PDIII and PDIV contain three NF‐κB binding sites, whereas PDII contains an ATF2/c‐Jun binding site (Fig. 3A). These regions lie within the 150 base pairs (bp) upstream of the transcriptional start site. To further explore the mechanisms by which E‐selectin is differentially regulated by WT, *lpxA‐* and LPS, two E‐selectin promoter constructs (tagged to the luciferase reporter gene) were used. The first construct contained 166 bp upstream of the start site (‐166) and contains the WT PDII promoter region (TGACATCATTG). The second construct contains a mutation within the PDII site (‐166M, Fig. 3A; gtcgAgCcTTG), which blocks ATF2 binding to this region (van Hooft *et al*., 1992) (‐166M; Fig. 3A).

Human umbilical vein endothelial cells (HUVEC) transfected with the E‐selectin promoter constructs were co‐cultured with WT or *lpxA‐* bacteria or stimulated with LPS, and luciferase activity quantified 6 h post‐stimulation. WT and *lpxA‐* meningococci induced luciferase activity upon activation of the ‐166 promoter (Fig. 3B and C). In contrast, purified meningococcal LPS induced lower levels of the promoter activity (Fig. 3D). These data further confirmed that WT, *lpxA‐* and purified LPS have the ability to differentially regulate E‐selectin transcription, consistent with the observed surface expression and mRNA levels (Figs 1 and 2). These studies suggested that the variation in E‐selectin expression observed in response to WT and *lpxA‐* bacteria and the purified LPS were due to differential activation of the E‐selectin promoter.

While WT induction of the ‐166M promoter was partially reduced compared with the ‐166 promoter construct, there was some residual activity (Fig. 3B), suggesting that the presence of NF‐κB sites was sufficient to induce low levels of the promoter activity. Induction of the ‐166M promoter by *lpxA‐* bacteria was markedly reduced compared with the 166 construct (Fig. 3C), leading us to conclude that the ATF2/c‐Jun binding element is critical to the E‐selectin promoter response to non‐LPS bacterial products. LPS‐induced promoter activity with the ‐166M mutant was also reduced (Fig. 3D); however, the overall percentage reduction was lower than with WT and *lpxA‐* bacterial stimuli (54% vs. 60% and 90% respectively). The markedly lower levels of promoter activity observed with the ‐166M construct compared with the ‐166 construct strongly suggested that E‐selectin promoter activity stimulated by WT and *lpxA‐* meningococci requires a functional PDII region for maximal expression. Collectively, the differential expression pattern observed on the surface (Fig. 1), mRNA (Fig. 2) and promoter activity level (Fig. 3) suggested that the LPS‐independent signalling mechanisms regulating E‐selectin promoter activity require the activity of the PDII region of the E‐selectin promoter.

### *N* *. meningitidis* H44/76 infection of primary endothelial cells mediates rapid phosphorylation of ATF2 transcription factor

ATF2 phosphorylation is an absolute requirement for the heterodimerization of ATF2 and the transcription factor c‐Jun and subsequent binding to PDII site in the E‐selectin promoter (van Hooft *et al*., 1992). To explore whether meningococci differentially affected AFT2 phosphorylation compared with LPS, ATF2 phosphorylation was investigated following stimulus with WT, *lpxA* and purified LPS by confocal microscopy.

**Figure 4:**
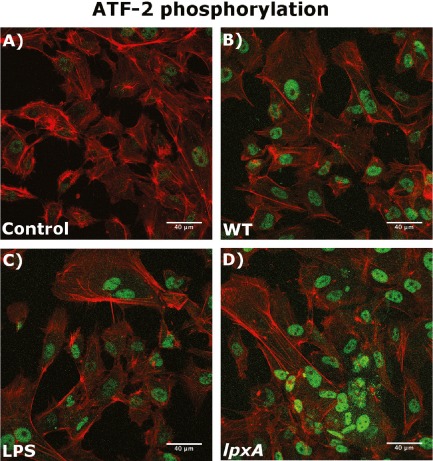
The *lpxA‐* bacteria induced higher levels of ATF2 phosphorylation than WT bacteria and purified LPS. Cells were grown on coverslips and kept in media only (A, Control), or stimulated for 30 min with 10^8^ cfu ml^−1^ WT (B) or *lpxA‐* (C) bacteria, 10 ng ml^−1^ purified LPS (D). HUVEC were then stained for actin (red) and phospho‐ATF2 (green) as described in the Experimental procedures section. Cells were visualized using a scanning laser confocal microscope.

**Figure 5:**
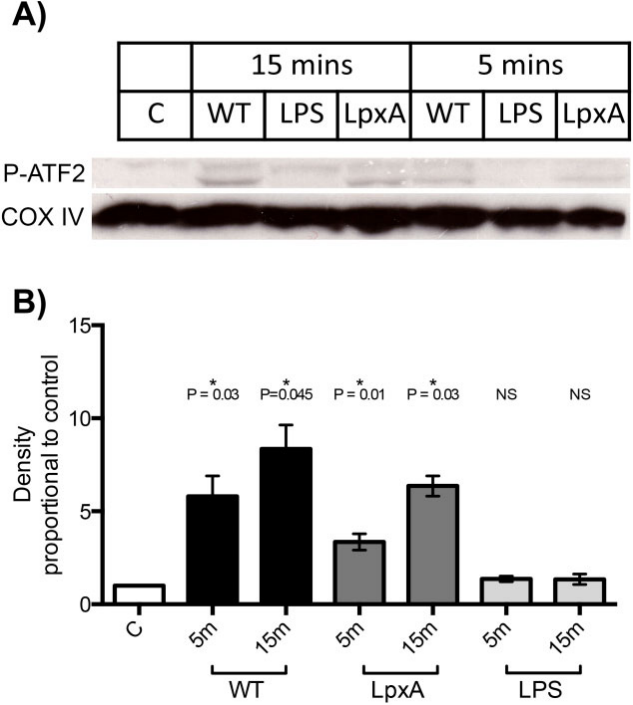
ATF2 phosphorylation is stimulated by WT and *lpxA‐* bacteria, but not LPS. A. Representative Western blot showing ATF2 phosphorylation in HUVEC after 15 min stimulation by 10^8^ cfu ml^−1^ fixed WT or *lpxA‐* bacteria, or 10 ng ml^−1^ LPS. COX IV antibody was used as a loading control. B. Summary of mean ATF2 phosphorylation presented as fold increase in band density above control ± SEM, *n* = 3.

ATF2 phosphorylation was undetectable in unstimulated, control HUVEC (Fig. 4A). There was minimal activation of ATF2 phosphorylation observed after 30 min of exposure to LPS (Fig. 4, panel C); however, in HUVEC stimulated with either WT or *lpxA* strains (Fig. 4B and D, respectively), phosphorylated ATF2 was clearly detectable within the nuclei. In fact, ATF2 phosphorylation was detectable within 5 min of exposure to WT or *lpxA‐* bacteria (data not shown). To confirm this finding, ATF2 phosphorylation was quantified using Western blot analysis (Fig. 5A). While both WT and *lpxA‐* bacteria induced significantly higher levels of phosphorylated ATF2 after 15 min of exposure, as compared to control (*P* = 0.03 and 0.01, respectively), there was no increase in ATF2 phosphorylation above background levels upon LPS stimulation at this time point (Fig. 5B). Both WT and *lpxA‐* bacteria produced significantly higher levels of phosphorylated ATF2 compared with the purified LPS (*P* = 0.04 and *P* = 0.01, respectively).

### The LPS‐deficient lpxA‐ is a weak inducer of nuclear NF‐κB translocation compared with WT bacteria and purified LPS

**Figure 6:**
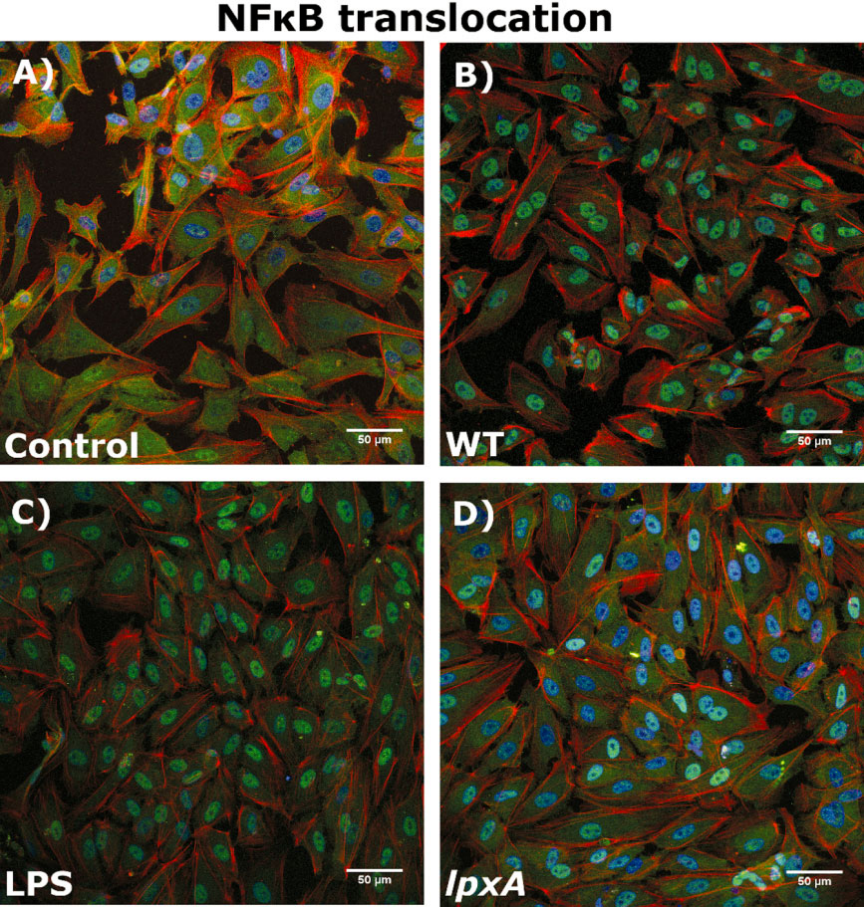
WT bacteria and purified LPS induce higher levels of NF‐κB translocation to the nucleus than *lpxA‐* bacteria. Cells were grown on coverslips and stimulated for 2 h with 10^8^ cfu ml^−1^ WT (B) or *lpxA‐* bacteria (C), 10 ng ml^−1^ purified LPS(D), or media only (Control, A). HUVEC were then stained for actin (red), DAPI nuclear stain (blue) and NF‐κB (green) as described in the Experimental procedures section. Cells were visualized using a scanning laser confocal microscope.

To investigate NF‐κB activity in response to LPS and non‐LPS *N. meningitidis* bacterial components, NF‐κB translocation and activation in response to the various stimuli were investigated by confocal microscopy. As shown in Fig. 6A, in unstimulated control HUVEC, NF‐κB was found to be localized in the cytoplasm. Upon infection with the WT bacteria, nuclear translocation of NF‐κB was clearly observable within 2 h post infection (Fig. 6B). A similar response was also seen in response to purified LPS (Fig. 6C). However, the *lpxA‐* bacteria showed reduced ability to induce NF‐κΒ nuclear translocation (Fig. 6D) as was seen by the lack of co‐localization in DAPI‐labelled (blue) nuclei in the *lpxA‐* stimulated HUVEC (compared with the WT and LPS‐stimulated cells, where all the nuclei are cyan). This indicated that the *lpxA‐* stimulated HUVEC resulted in weak NF‐κB activation, compared with the purified LPS of WT bacteria, supporting our previous observation that the meningococcal LPS‐dependent pathway is a major regulator of the NF‐κB activity (Dixon *et al*., 2004).

### *N* *. meningitidis* H44/76 LPS‐independent mechanisms of E‐selectin expression are predominantly regulated through the MAP kinase family

Members of the MAPK family p38 and JNK kinases are upstream activators of ATF2 phosphorylation in endothelial cells (Min and Pober, 1997; Read *et al*., 1997; Jersmann *et al*., 2001). In order to explore the role of the MAPK pathways in the regulation of E‐selectin expression by *N. meningitidis*, E‐selectin surface expression was determined in the presence of p38 and JNK inhibitors (SB203580 and SP600125, respectively). An NF‐κΒ inhibitor (MG132) was used as a control.

**Figure 7:**
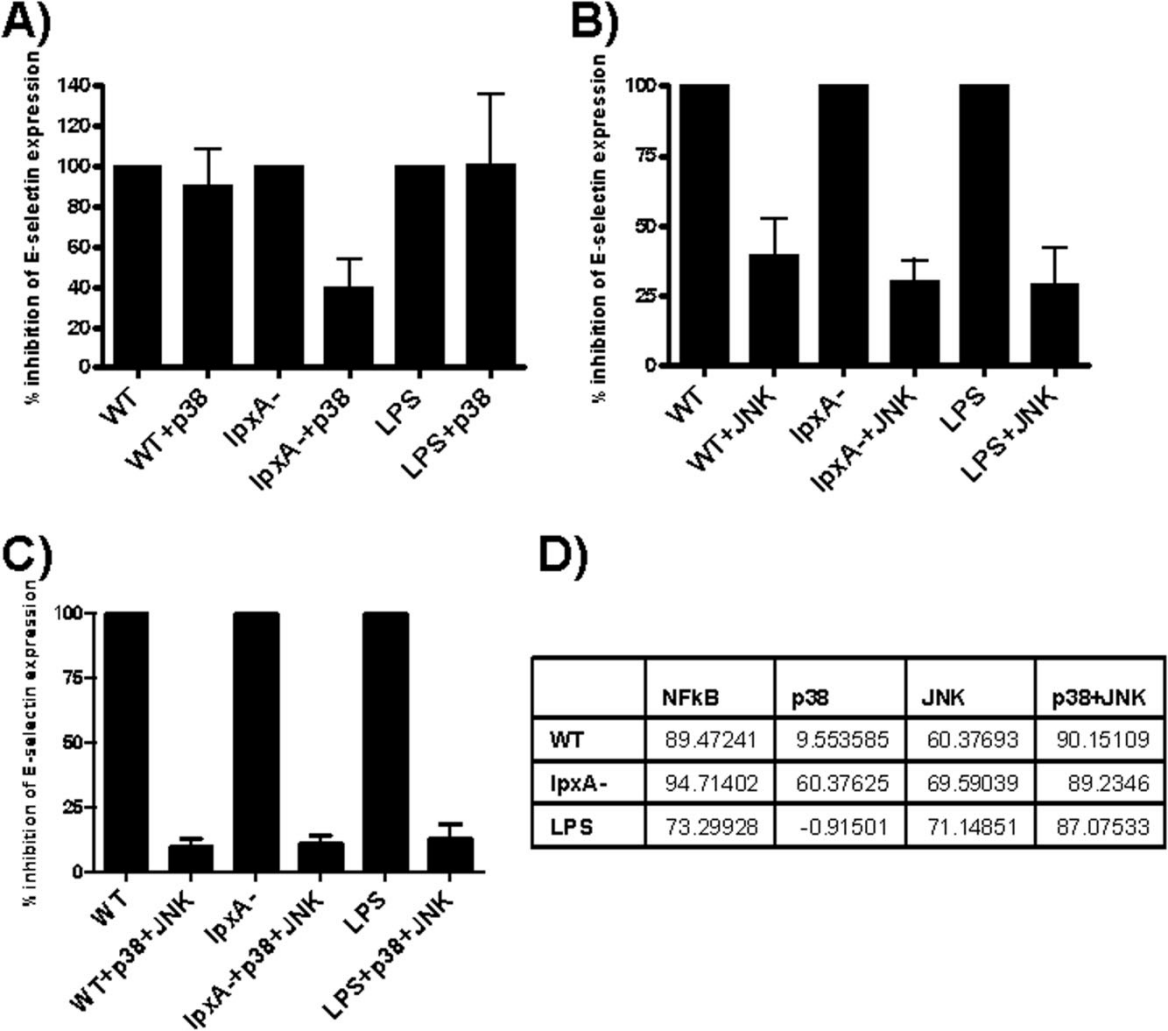
Inhibition of E‐selectin expression by p38 and JNK MAPK pathway inhibitors, Inhibitors were added 30–60 min prior to 5 h stimulation with 10^8^ cfu ml^−1^ WT and *lpxA‐* bacteria or 10 ng ml^−1^ purified LPS. E‐selectin surface expression was detected by FACS analysis, as described in the Experimental procedures section. (A) p38 inhibition, (B) JNK inhibition, (C) p38 and JNK combined inhibition. Results are expressed as MFI E‐selectin expression ± SEM percentage inhibition of the MFI of E‐selectin expression. *n* = 4. (D) Table expressing percentage inhibition in E‐selectin expression induced by WT, *lpxA‐* and LPS by the three inhibitors.

The presence of a p38 inhibitor had no effect on E‐selectin expression in response to WT and LPS stimulation (Fig. 7A). In contrast, inhibition of p38 kinase had a significant effect on *lpxA‐* mediated E‐selectin expression, resulting in an ∼ 60% reduction in E‐selectin expression (Fig. 7A and D, *P* = 0.02), suggesting that E‐selectin expression induced by the *lpxA‐* bacteria was more dependent upon p38 kinase activation than WT and LPS.

In contrast, inhibition of JNK resulted in 60–70% reduction in E‐selectin expression in response to all the three stimuli (Fig. 7B). Taken together, the data indicated that p38 may play a predominant role in LPS‐independent stimulation, whereas JNK may be more crucial for LPS‐dependent E‐selectin expression during *N. meningitidis* infection.

The use of both the p38 and the JNK inhibitors together completely inhibited E‐selectin expression in response to all three stimuli (∼90%; Fig. 7C). This demonstrated that the E‐selectin promoter absolutely requires MAP kinase activation to induce E‐selectin expression on endothelial cells in response to both LPS‐dependent and LPS‐independent *N. meningitidis* components.

## Discussion

Early signalling events initiated by contact of both bacteria and their products with vascular endothelium are likely to have a major influence on regulating endothelial physiology, inflammation and subsequent leucocyte behaviour. These processes will in turn have a profound effect on the course and severity of bacterial sepsis, exemplified by infections caused by the gram‐negative bacterium *N. meningitidis*. Of these signals, LPS, contained within the meningococcal outer membrane, has historically been considered to be the most potent mediator of endothelial inflammatory responses (Heyderman *et al*., 1997) and circulating level of LPS in the host is a strong predictor of meningococcal disease outcome (Brandtzaeg *et al*., 1989). However, work from our laboratory and others show that meningococcal components other than LPS can deliver additional signals to endothelial cells that may in part explain the capacity of bacteria to inflict the vascular damage that is so characteristically seen in most severely affected patients (Klein *et al*., 1996; Dixon *et al*., 1999; 2004; Harrison *et al*., 2002).

We have previously demonstrated that live LPS‐deficient bacteria, *lpxA‐*, is able to induce high levels of endothelial cell adhesion molecule expression, particularly E‐selectin and that this is achieved despite the *lpxA‐* strain being a very weak activator of the major transcription factor NF‐κB (Dixon *et al*., 2004). In this present study, we demonstrated that killed bacteria were able to induce adhesion molecule surface protein expression as well as up‐regulation of mRNA transcription (Figs 1 and 2), suggesting that E‐selectin regulation by bacteria occurs at the transcriptional level, as seen by other E‐selectin inducers (Montgomery *et al*., 1991; Scholz *et al*., 1996; Wyble *et al*., 1997).

We chose to focus on the E‐selectin promoter function to determine the relative contributions of LPS and non‐LPS components in driving transcription of this important cell adhesion molecule. The results presented in this study show that WT, purified LPS and LPS‐deficient bacteria induce E‐selectin promoter activity, resulting in transcriptional activity that mirrors the differential capacity of these different stimuli to induce E‐selectin surface expression. The data also show that the capacity of the *lpxA‐* bacterium, and therefore non‐LPS components, to drive E‐selectin promoter activity is dependent upon an intact PDII site (Fig. 3).

It is known that the PDII region is critical for E‐selectin promoter activity, as the mutation contained in the ‐166M promoter construct abolishes ATF2 binding to this site (van Hooft *et al*., 1992) and reduces E‐selectin promoter activity in response to cytokine (Interleukin 1 β and Tumor Necrosis Factor‐α) stimulation (van Hooft *et al*., 1992; Kaszubska *et al*., 1993; Schindler and Baichwal, 1994; Tamaru and Narumi, 1999). Therefore, the likely explanation for the capacity of whole meningococci to induce E‐selectin promoter activity involves their capacity to induce phosphorylation of ATF2 and subsequently activating this transcription factor on the PDII site. The results presented here show that meningococci, in contrast to purified LPS, are potent inducers of ATF2 phosphorylation in primary endothelial cells. ATF2 is known to be phosphorylated via the action of a number of upstream kinases, including p38 and JNK MAP kinases (Read *et al*., 1997). It is interesting to note that inhibition of p38 kinase results in reduction of E‐selectin expression only in response to the *lpxA‐* bacteria, but not to purified LPS and WT bacteria. While the underlying reasons behind this observation are not clear at this time, it has previously been demonstrated that WT bacteria and LPS primarily activate NF‐κB, most likely via the TLR4‐MyD88 signalling pathway (Pridmore *et al*., 2001; 2003). The *lpxA‐* bacteria may be using other TLRs, such as TLR2 (Pridmore *et al*., 2001), to direct gene activation via the MAP kinase pathway; however, it remains unclear if these mechanisms are involved in endothelial cell responses to *N. meningitidis*. We propose from this study that the predominant signals from LPS‐independent components of meningococci are via the MAP kinase pathway, and in the case of *lpxA* strain, p38 kinase resulting in ATF2 phosphorylation. However, the weak induction of NF‐κB by non‐LPS components is still an absolute requirement for E‐selectin expression.

Previous studies have demonstrated that *N. meningitidis* can activate the MAPK signalling pathway, causing phosphorylation of JNK1 and 2 and p38 (Sokolova *et al*., 2004). In HUVEC, the higher levels of ATF2 phosphorylation induced by MAP kinases may be important in E‐selectin promoter activity in situations where low levels of NF‐κΒ activation are present. Studies have shown that NF‐κΒ and ATF2 not only associate with the promoter sequence but are also able to interact with each other (Kaszubska *et al*., 1993). This interaction may cause enhanced promoter activity, but the precise mechanisms by which this takes place are still to be defined.

It is not yet clear which host pattern recognition receptors (PRRs) are involved in meningococcal‐induced E‐selectin promoter activation; however, they are likely to involve TLRs, particularly TLR 2 and TLR4. Endothelial cells express PRRs on the cell surface, and both these receptors are able to activate the NF‐κB and MAPK pathways (Re and Strominger, 2001; Asehnoune *et al*., 2005) on dendritic cells and neutrophils. WT meningococci have been shown to signal through TLR2 and 4 on human macrophages, monocytes, and peripheral blood mononuclear cell as well as in mouse model systems (Pridmore *et al*., 2001; 2003; Mogensen *et al*., 2006a, 2006b), whereas the *lpxA‐* mutant meningococci and *Neisserial* porB have both been shown to signal through TLR2 alone or in combination with TLR1, respectively, on human macrophages and cell lines (Schwandner *et al*., 1999; Pridmore *et al*., 2001; Massari *et al*., 2002; 2006; Hellerud *et al*., 2008).

Of more interest and relevance to this study are the relative potencies of LPS/TLR4‐driven signals and those that stem from activation by TLR2. Our results are in agreement with the findings of Hellerud *et al*., who found that LPS‐independent signals appear only to induce significant inflammatory responses at high multiplicity of infection, specifically at ranges above 10^7^ cfu ml^−1^ (Hellerud *et al*., 2008). These data indicate that a critical threshold may exist whereby LPS‐independent components of meningococci induce equivalent inflammatory responses to LPS‐mediated responses, and the data presented here in relation to E‐selectin expression, transcriptional and promoter activity would appear to support that notion. This is of direct biological and clinical significance since these levels of bacterial load in circulating plasma of patients are associated with massive and uncontrolled inflammation, severity of disease and poor outcome (Ovstebo *et al*., 2004). There has been little success in the use of anti‐endotoxin therapies in reducing mortality in both adults and neonates (Alejandria *et al*., 2013), possibly due to the non‐LPS‐mediated signals that occur at high bacterial loads. This suggests that the mortality observed in response to meningococcemia could be exacerbated by the non‐LPS components of the bacteria and may be a target for future therapies.

In addition to TLR2/4‐mediated signals, it is possible that *Neisserial* adhesins, including pili and opacity proteins, could activate MAP kinases independent of TLR signalling, which could act in concert with TLR4 and 2 signalling to modulate E‐selectin expression via activation of either JNK or p38 MAP kinase. However, E‐selectin induction in HUVEC has been found to be no different in response to pileated WT and non‐adherent pilus‐deficient mutants (Linhartova *et al*., 2006), suggesting that pilus‐mediated adhesion is unlikely to be a significant mechanism governing E‐selectin expression, at least in primary endothelial cells. It is unknown whether downstream signalling induced by Opa could influence E‐selectin promoter activity. Opa from *Neisseria gonorrhoeae* (NG) has been demonstrated to bind to newly expressed carcinoembryonic antigen‐related cellular adhesion molecules (CEACAMs) (Muenzner *et al*., 2001), leading to increased internalization of NG (Muenzner *et al*., 2008). Further work is required to address this potential mechanism. However, as CECAMs are not constitutively expressed on HUVEC, but require additional activation signals (by bacteria themselves) to be up‐regulated (Muenzner *et al*., 2001), it is unclear whether this mechanism could fit with the short timescale involved in ATF2 phosphorylation observed in this study and *de novo* E‐selection transcriptional responses.

The other finding highlighted by our study is that in contrast to our initial hypothesis, enhanced E‐selectin promoter activity, and consequently E‐selectin expression, does not appear to depend on whether meningococci are viable or killed. Potentially, mechanisms initiated by metabolically inactive organisms could also provide additional signals that could induce MAP kinase activity and ATF2 phosphorylation. Consequently, it may not be just an initial insult with live bacteria, but a continuous activation and re‐activation of the immune system by killed bacteria. While it has recently been demonstrated that bacterial adhesion to the human endothelium is critical for the development of vascular damage observed in meningococcal disease (Melican and Dumenil, 2012; Join‐Lambert *et al*., 2013; Melican *et al*., 2013), our study suggests that killed bacteria may also play a role in mediating and maintaining the continuous vasculopathy and coagulopathy seen during meningococcal sepsis. Clearance or inhibition of any bacterial products that include, but are not limited to, LPS from the blood stream could be of primary importance in managing meningococcal sepsis.

The challenge consequently is to further understand how these early signalling events ultimately control leucocyte adhesion and activation that is part of the mechanism of vascular damage characteristic of severe disease. These data also further support the growing body of evidence that strategies that target LPS/TLR4 pathways alone may not be effective in situations where there is high bacterial loads and potentially ongoing signalling by adherent bacteria. Alternative treatment approaches that target multiple mechanisms, such as non‐LPS components of the bacteria as well as the MAP kinases, need to be considered.

## Experimental procedures

### Reagents

Lipopolysaccharide was purified from *N. meningitidis* H44/76 (immunotype L3, 7, 9) by hot aqueous phenol extraction, ultracentrifugation, gel filtration and cold ethanol–NaCl precipitation, as previously described (Andersen *et al*., 1995). Antibodies: Anti‐Phospho‐ATF2 (Thr71) (Cell Signaling Technology, MA, USA), Anti‐COX IV (Cell Signaling Technology), horseradish peroxidase conjugated goat anti‐rabbit (Cell Signaling Technology).

### Bacterial strains

Bacterial strains used in this study were developed and provided by Dr. Peter van der Ley (RIVM, Bilthoven) and Dr. Liana Steeghs (University Medical Centre Utrecht, the Netherlands). The *N. meningitidis H44/76* WT strain used in this study is referred to as H44/76 [sero (sub)‐type (B:15:P1.7,16), ET‐5 complex]. This strain was isolated from a fatal human septicaemia case in Norway (Holten, 1979). A viable LPS‐free isogenic mutant (*lpxA‐*) of the H44/76 WT strain was developed by inactivating the *lpxA‐* gene with a kanamycin cassette (Steeghs *et al*., 1998). Bacteria were grown from frozen stock in freshly prepared Gonococci (GC) agar plates supplemented with Vitox growth medium, and kept at 35°C in 5% CO_2_ in air. Bacteria were collected at the stationary phase for experiments. PFA‐killed bacteria were prepared by collecting several colonies of bacteria with a sterile cotton swab and suspended in warm RPMI (without phenol red) supplemented with 0.5% PFA. Optical density of the bacterial suspension was measured by spectrophotometry at an OD_540 nm_, and the bacterial suspension was adjusted to give a reading of 1.

### Cell culture

Preparation of endothelial cells from umbilical cords was performed by collagenase type 2 (Invitrogen) digestion, as previously described by Kotowicz *et al*. (2000). For the growth of HUVEC, MCDB 131 was supplemented with 10 mM L‐glutamine, 100 units ml^−1^ penicillin/streptomycin, 0.25 μg ml^−1^ Amphotericin B and 20% FCS (20% MCDB). HUVEC were grown to 95% confluence in 25 cm^2^ flasks. Verification of endothelial cell culture was determined both morphologically and phenotypically by CD31 surface staining. HUVEC were passaged by detaching the cells using Accutase (PAA Laboratories). HUVEC were seeded onto culture plates coated with attachment factor (TCS Cell Works) and grown to confluence over 3–7 days in 20% MCDB. Once the cells had reached confluence, they were rested in RMPI‐1640 supplemented with 10 mM L‐glutamine, 100 units ml^−1^ penicillin/streptomycin, 0.25 μg ml^−1^ Amphotericin B and 20% FCS (20% RPMI). For E‐selectin promoter transfection experiments, HUVEC were seeded on attachment factor‐coated culture plates and grown in 20% MCDB supplemented with endothelial cell growth serum (ECGS; PromoCell). HUVEC were used at passage 1 for all experiments described.

### Immunofluorescence and flow cytometry

Adhesion molecule expression was detected as previously described (Dixon *et al*., 1999). Briefly, cells were incubated with mouse monoclonal antibodies to human E‐selectin (CD62E), ICAM‐1 (CD54) and VCAM‐1 (CD106) (Serotech, Oxford, UK) followed by goat anti‐mouse F(ab′)_2_‐phycoerythrin conjugate (Dako). Non‐specific binding of antibodies was controlled for by inclusion of an irrelevant isotype‐matched control (Dako). Samples were washed and resuspended in Cellfix (Becton Dickinson), and analysed by flow cytometry (FACScalibur, Becton Dickinson) with Cell Quest software (Becton Dickinson). The endothelial cell population was identified by its forward‐scatter and side‐scatter position and by expression of CD31 (Serotec). For each sample, 5000 events were collected within the endothelial gate. Median fluorescence intensity (MFI) was calculated by FlowJo software on the gated endothelial cells, only.

### mRNA extraction and real‐time PCR

mRNA was extracted using the RNeasy Protect Mini‐Kit (Qiagen), and the protocol was followed according to manufacturer's instructions. Total RNA was eluted in 30 μl of nuclease‐free H_2_O and stored at −80°C. About 5 μg of total RNA was used for cDNA synthesis using iScript cDNA synthesis kit (BioRad) in a final volume of 20 μl, using the manufacturer's instructions.

For real‐time PCR reactions, cDNA template was amplified using iQ SYBR Green Supermix (BioRad), following manufacturer's instructions with some modifications. Briefly, reactions were carried out in 10 μl volumes using 1 μl template cDNA in 1× iQ SYBR Green Supermix. Primers were added at a final concentration of 0.5 μM. Reactions were performed in duplicate using the MJ Opticon Real‐Time PCR instrument (GRI). cDNA was initially denatured at 95°C for 15 min, followed by 40 cycles of denaturation: 95°C for 30 s annealing, 60°C, 30 s extension, 72°C, 30 s; melting of DNA, 79°C, 10 s; fluorescence measurement, 79°C, 1 s. At the end of the assay, melt‐curve analysis was performed. Primers used for real‐time RT‐PCR were as follows: Cyclophilin (F): 5′‐GTCAGCAATGGTGATCTTCTT‐3′, (R): 5′‐GCAGAAAAT TTTCGTGCTCTG‐3′ (Samady *et al*., 2004); E‐selectin (F): 5′‐GGGAATTCGT GTGACCCTGGCTTC‐3′, (R): 5′‐GGAAGCTTGGAATAGGAGCACTCC‐3′; VCAM‐1 (F): 5′‐ATGACATGCTTGAGCCAGG‐3′, (R): 5′‐GTGTCTCCTTC TTTGACACT‐3′ (Zhang *et al*., 2002).

Data were normalized using a mathematical method (referred to here as the gene induction method). Briefly, the technical duplicate values were averaged for each gene. To obtain the induction of the gene of interest, the average expression of the gene of interest was normalized to the average expression of the housekeeping control gene.

### Reporter gene assays

Endothelial cells were transfected as previously described using the LID vector system, with some amendments (Hart *et al*., 1998; Writer *et al*., 2004). Briefly, the LID complex was prepared by combining Lipofectin (Life Technologies, UK) with Peptide 6 (peptide sequence: [K]_16_‐GACRRETEWACG) (Hart *et al*., 1998; Writer *et al*., 2004) at a final peptide : DNA ratio of 7:1. Finally, DNA plasmids were added to the transfection cocktail: 210 ng of the Picagene plasmid (containing the E‐selectin promoter) and 40 ng of the phRL‐TK plasmid (expressing the Renilla luciferase for a positive transfection control). The transfection cocktail was prepared in OptiMEM‐1 reduced serum (Life Technologies, UK) and supplemented with 2.5 mM sodium butyrate (SB). Cells were plated at 3 × 10^4^ per well in a 96‐well plate the previous day, were aspirated, and the transfection mix was added shortly after making the LID complex. The transfection cocktail was left on the cells for 4 h at 37°C 5% CO_2_ in air and was then replaced with fresh 10% MCDB supplemented with ECGS (PromoCell), epidermal growth factor (R&D Systems) and 2.5 mM SB (Sigma). The day after transfection, cells were stimulated for 5 h with 10^8^ cfu ml^−1^ WT and *lpxA‐* bacteria, 10  ng ml^−1^ purified LPS, or 400 gp ml^−1^ IL1β and 100 ng ml^−1^ PMA simultaneously (IP). All conditions were carried out in replicates of 6 wells each. Transfection reactions were terminated by transferring cells to ice and washing cells twice with ice‐cold 1× phosphate‐buffered saline (PBS). Cells were harvested in 50 μl 1× reporter lysis buffer (Luciferase Assay System–Promega) and kept at −80°C. Luciferase assays were performed using the luciferase assay system (Promega), following the manufacturer's instructions, on white polystyrene, 96‐well luciferase assay plates (Porvair Sciences). Assays were performed on a Luci‐1 Luminometer (Anthos, Salzburg, Austria). Transfections were performed with a control plasmid expressing Renilla luciferase. This was used to normalize luciferase activity according to the transfection efficiency for each individual well. Renilla substrate was prepared by diluting 1 mg of Coelenterazine (Promega) in 1 ml of ethanol and 20 μl aliquots was stored at −80°C. For Renilla assays, Coelenterazine was used in a 1:500 dilution in 1× PBS. Renilla assay was performed as the luciferase assay described above. Luciferase activity was expressed as relative luciferase units (RLU)/relative Renilla units (RRU).

### Inhibition studies

Human umbilical vein endothelial cells were pre‐incubated with specific inhibitors (Calbiochem, Nottingham, UK) for 30–60 min prior to and during stimulation with PFA‐killed bacteria or purified LPS. Inhibitors used in these studies were SB203580 (p38) and MG132 (NF‐κB) at 25 μM and SP600125 (JNK) at 50 μM. Inhibitors were kept on the cells throughout the experimental procedure.

### Confocal microscopy

For confocal microscopy analysis of ATF2 phosphorylation and NF‐κB translocation to the nucleus, HUVEC grown on coverslips were incubated with *N. meningitidis H44/76* or purified LPS for 30 min or 2 h. Cells were washed three times with sterile PBS and fixed with 4% paraformaldehyde for 30 min. Cells were permeabilized for 30 min in PBS with 0.5% bovine serum albumin (BSA) supplemented with 0.1% Triton X‐100 (Sigma), and non‐specific binding of antibody was blocked by incubating cells with 0.5% BSA for 30 min. Cells were visualized by staining the nuclei with To‐Pro3 and Rhodamine‐Phalloidin (Molecular Probes, Cambridge Biosciences, Cambridge, UK). NF‐κB and phosphor‐ATF2 were visualized using an unlabelled rabbit anti‐NF‐κB p65 antibody or unlabelled mouse anti‐pATF2 antibody (both from Santa Cruz Biotechnology) followed by incubation with a FITC‐conjugated goat anti‐rabbit IgG antibody (Sigma) or goat anti‐mouse IgG antibody, respectively, for 30 min. Slides were then washed and mounted with Vectashield (Vector laboratories, Peterborough, UK) and images were obtained using a Leica SP2 confocal laser scanning microscope system (Leica, Milton Keynes, UK). Confocal images were processed using the open source image processing software Fiji ImageJ (Schindelin *et al*., 2012).

### Western blotting

Human umbilical vein endothelial cells were incubated with *N. meningitidis* bacteria or purified LPS for 15 min. Cells were washed with ice‐cold PBS and then lysed with Laemmli buffer (2% sodium dodecyl sulfate (SDS), 5% 2‐mercaptoethanol, 10% glycerol, 0.002% bromophenol blue, 1% Triton X‐100, 0.63 M Tris–HCL). Cell lysates were incubated on ice for 20 min, passed through a 23 gauge needle and frozen at −80°C. Samples were thawed and then boiled for 20–30 min. Lysates were run on 12% polyacrylamide gels under denaturing conditions. Proteins were transferred onto Polyvinylidene Difluoride (PVDF) membrane (Amersham Hybond‐P, GE Healthcare, Buckinghamshire, UK) and blocked with 5% milk powder (Marvel) in 0.1% Tween‐PBS. Membranes were incubated overnight at 4°C with primary antibodies against P‐ATF2 (1:1000) and Cox‐IV (1:3000), followed by secondary HRP‐conjugated anti‐rabbit antibody (1:2000) at room temperature for 1 h. Proteins were visualized using ECL detection kit (Amersham), and comparative staining densities were calculated using ImageJ and the gel analysis software (Miller, 2010).

### Statistical analysis

Data were analysed using one‐way analysis of variance (ANOVA) with Bonferroni's post‐test correction, using GraphPad Prism version 5.03 for Windows (GraphPad Software, San Diego, CA, USA, http://www.graphpad.com).
